# De Novo Analysis Reveals Transcriptomic Responses in *Eriobotrya japonica* Fruits during Postharvest Cold Storage

**DOI:** 10.3390/genes9120639

**Published:** 2018-12-17

**Authors:** Shoukai Lin, Ti Wu, Hailan Lin, Yanqing Zhang, Shichang Xu, Jinge Wang, Bisha Wu, Yu Chen, Suying Lin, Dahe Lin, Xiumei Wang, Xiaoxu Zhao, Jincheng Wu

**Affiliations:** 1 Key Laboratory of Loquat Germplasm Innovation and Utilization (Putian University), Fujian Province University, Putian 351100, China; linshoukai@ptu.edu.cn (S.L.); linhailan.ok@163.com (H.L.); zyq190318@outlook.com (Y.Z.); 17720837915@163.com (S.X.); 3170515028@fafu.edu.cn (J.W.); wubisha@163.com (B.W.); chenyu@ptu.edu.cn (Y.C.); linsuying@ptu.edu.cn (S.L.); dahelin01@163.com (D.L.); wxm890626@163.com (X.W.); 2 Fujian Provincial Key Laboratory of Ecology-toxicological Effects & Control for Emerging Contaminants, Putian University, Putian 351100, China; zhaoxiaoxu@ptu.edu.cn; 3 Faculty of Agriculture, Dalhousie University, Truro, NS B2N 5E3, Canada; tz983708@dal.ca; 4 Overseas Education College, Fujian Agriculture and Forestry University, Fuzhou 350002, China; 5 College of Life Science, Fujian Agriculture and Forestry University, Fuzhou 350002, China

**Keywords:** loquat fruit, transcriptomic responses, lignification, postharvest cold storage, RNA-seq, gene expression, signal transduction, sugars and polysaccharides metabolism

## Abstract

Cold storage is the primary preservation method of postharvest loquat fruits. However, cold storage also results in many chilling injury physiological disorders called lignification, which decreases the quality and economic value of the fruits. Few studies to date have focused on the transcriptomic responses associated with lignification except lignin synthesis pathways. This study aimed to explore the changes of loquat transcriptome during long-term cold storage. Our results showed that the gene expression patterns were differed among the five stages. The differentially expressed genes (DEGs) in response to cold storage were more intense and complex in earlier stage. The membrane-related genes preferentially responded to low temperature and were followed by intracellular-located genes. The cold-induced pathways were mainly concerned with signal transduction and secondary metabolism (i.e., lignin, pectin, cellulose, terpenoid, carotenoid, steroid) in the first three stages and were chiefly related to primary metabolism in the later two stages, especially energy metabolism. Further investigation suggested that 503 protein kinases, 106 protein phosphatases, and 40 Ca^2+^ signal components were involved in the cold signal transduction of postharvest loquat fruits. We predicted a pathway including 649 encoding genes of 49 enzymes, which displayed the metabolisms of major sugars and polysaccharides in cold-stored loquat fruits. The coordinated expression patterns of these genes might contribute to the changes of saccharides in the pathway. These results provide new insight into the transcriptomic changes of postharvest loquat fruits in response to cold storage environment, which may be helpful for improving the postharvest life of loquat in the future.

## 1. Introduction

Loquat (*Eriobotrya japonica* Lindl.) is a subtropical and evergreen fruit tree native to China. Its fruits both have edible value for supplying luscious tastes and abundant nutrients and medicinal value for relieving cough and asthma in traditional Chinese medicine [1]. Cold storage is the primary preservation method of loquat fruits to effectively control the microbial-induced decay and nutritional loss after harvest, thereby prolonging the period for fresh eating and processing. However, cold storage also results in many chilling-injury phenomena, including stuck peel, hard texture, crude mouthfeel, less juice, weight loss, internal browning, and weak flavor. It is called lignification and decreases the quality and economic value of the fruits [2,3,4]. Unlike softening, fruit lignification was rarely reported in other cold storage fruits and loquat fruit has become the model for researching fruit lignification.

Many studies indicated that lignification was a series of fruits physiological disorders caused by chilling injury. The most obvious characteristics of loquat lignification were the increased hardness and loss of juiciness of fruit pulps which were mainly caused by the abnormal changes in cell wall metabolism. Cell walls are essentially composed of lignin, cellulose, hemicellulose, and pectin. Lignin could support and strengthen the cell wall. The activities of lignin synthesis related enzymes L-phenylalanine ammonia-lyase (PAL), cinnamate 4-hydroxylase (C4H), 4-coumarate:coenzyme A ligase (4CL), cinnamyl alcohol dehydrogenase (CAD) and peroxidase (POD) were sustained growth and promoted the accumulation of lignin [5,6,7]. The cell wall polysaccharides changes in loquat fruits were opposite to typical depolymerization during fruit softening, which exhibited decreasing levels of water- and cyclohexane-diamine-tetraacetic acid (CDTA)-soluble pectins and increasing contents of Na_2_CO_3_-soluble pectin, hemicellulose and cellulose. The activities of polygalacturonase (PG) and pectin methylesterase (PME) were inhibited by cold storage that contributed to the pectin changes [5]. The fruit flavor was determined by the ratio of sugar and organic acid. Sucrose, fructose, and glucose are the major sugars in loquat fruits, which were coordinately regulated by the sugar metabolism enzymes and led to the decreasing of total sugar in cold-stored loquat. Malic acid is the predominant acid in loquat fruits. The rapid decline of malic acid content was the main reason of the sharp decline of total organic acid in cold-stored loquat. The loss of total organic acid was greater than that of total sugar, which results in the weak flavors of cold-stored loquat fruits [8,9,10].

The expression of lignin synthesis related enzyme genes and their transcriptional regulation mechanism were well explored in cold-stored loquat fruits. Cold storage induced the expression of *EjCAD1* gene which was correlative to lignin accumulation [11]. The expression of *Ej4CL1* was sensitive to low temperature [6]. *EjCCoAOMT* was greatly up-regulated in the earlier stage of cold storage [1]. The encoding genes of transcription factors were also induced by cold storage to regulate the expression of down-stream genes. The expression of transcription activators such as *EjMYB1*, *EjMYB8,* and *EjNAC1* were enhanced by cold storage and reduced by heat or low temperature conditioning (LTC) treatment [12,13,14], while the expression of transcription repressors such as *EjMYB2*, *EjAP2-1*, *EjNAC3,* and *EjNAC4* was opposite [12,15,16]. Further research found that these transcription factors could regulate the enzymes of lignin synthesis in cold-stored loquat fruits. EjMYB1 and EjMYB2 participated in the transcriptional regulation of lignin synthesis and acted as transcription activator and repressor respectively [12]. EjMYB8 could interact with the promoter of *Ej4CL1*, thereby activating the transcription of *Ej4CL1* [14]. EjODO1 could activate the transcription of lignin synthesis related genes *EjPAL1*, *Ej4CL1,* and *Ej4CL5* to promote the lignin synthesis [17]. EjNAC1 could activate the promoter of *EjPAL1* and *Ej4CL1* [13] while EjNAC3 could activate *EjCAD1* [16]. EjAP2-1 regulated loquat lignification through interacting with *EjMYB1/2* to fulfill suppression function [15]. EjHSP3 could also interact with EjAP2-1 to coordinately regulate lignin synthesis [18]. In addition, some signaling related genes were reported to be response to cold storage, including ethylene signaling pathway related genes such as *EjETR1*, *EjCTR1,* and *EjEIL1* [19] and G protein genes such as *EjROP1.2* and *EjLGA1* [2,20].

The RNA-seq technology and de novo analysis are suitable omics strategy for the transcriptomic research of a plant with or without reference genome [21]. It has been applied to the research of postharvest storage of many fruits, including apple [22], peach [23], nectarine [24], citrus [25], and mango [26]. The transcriptomic analysis provided very valuable information that may be related to the tolerance or adaptation of these fruits to chilling. Therefore, many postharvest biology related pathways could be conveniently studied based on transcriptomic data, such as phenylpropanoid biosynthesis, starch and sucrose metabolism, and amino sugar and nucleotide sugar metabolism. However, transcriptomic information for postharvest loquat fruits in response to cold storage is still very limited to date except lignin synthesis related genes and several signaling related genes. Therefore, the RNA-seq technology and de novo analysis strategy would be carried out to investigate the transcriptomic responses of loquat fruits during cold storage. According to the functional prediction, we subsequently focused on genes related to cold signal transduction, including protein kinases, phosphatases, and Ca^2+^ signal components. Additionally, we also predicted a putative pathway related to the metabolisms of major sugars and polysaccharides in cold-stored loquat fruit.

## 2. Materials and Methods

### 2.1. Plant Materials

The loquat cultivar ‘Jiefangzhong (JFZ)’ was used in the present study. Three uniform mature loquat trees (approximately 10 years old) were selected from outdoor nursery in 25° 45′ N and 118° 55′ E (Changtai town, Putian city, Fujian province, China) and managed with common cultivation. When the loquat trees were in full bloom, redundant flowers were removed except full-blooming flowers which was defined as zero d after full-blooming (DAF). When fruits reached at 40 DAF, redundant fruits were removed except four uniform fruits in each cluster. Each cluster was then bagged to protect the fruits until the fruits were mature (125 DAF). Three biological replicates were collected from three mature loquat trees, and each replicate was mixed from thirty bagged fruit clusters and divided into six groups. Mature fruits were then stored at 4 °C and sampled one group at zero day, seven days, 14 days, 21 days, 28 days and 35 days of cold storage. Fruit samples were rapidly peeled and seeded before being frozen in liquid nitrogen. Frozen pulps were mechanically ground and quickly blended in liquid nitrogen cooling appliances, and then stored at −80 °C.

### 2.2. RNA Extraction, cDNA Library Construction and Illumina Sequencing

Total RNA was extracted using the E.Z.N.A.™ Plant RNA Kit (Omega, Norcross, CA, USA) with DNase I digestion from each plant sample according to the manufacturer’s protocol. The quality of RNA was detected using Agilent 2100 Bioanalyzer (Agilent, Santa Clara, CA, USA) and the RIN number of RNA should be more than 7.0. Eligible RNA samples were enriched by removing rRNA using Ribo-Zero^TM^ Magnetic Kit (Epicentre, Madison, WI, USA) and subsequently used for cDNA library construction using NEB Next Ultra Directional RNA Library PrepKit for Illumina (New England Biolabs, Ipswich, MA, USA), following the manufacturer’s protocol. The cDNA libraries were sequenced using Illumina HiSeq^TM^ 4000 (Illumina, San Diego, CA, USA) to generate paired-end 150 bp (PE150) reads by Gene Denovo Biotechnology Co. (Denovo Biotechnology, Guangzhou, China).

### 2.3. Transcriptome Assembly and Unigene Annotation

Raw reads were filtered to remove reads containing adapters, unknown nucleotides (more than 10% of N) and low-quality reads (more than 40% of Q ≤ 20 bases) and the filtered reads were high quality clean reads. The Q20 (proportion of nucleotides with quality value larger than 20), Q30 (proportion of nucleotides with quality value larger than 30), and GC-content of obtained clean reads were evaluated. Transcriptome de novo assembly was carried out by Trinity program with default parameters to assemble the high quality clean reads into unigenes [21]. Assembly quality was assessed by length distribution statistics of unigenes. The functions of unigenes were annotated using Basic Local Alignment Search Tool (BLAST) program [27] (E-value cut-off = 1 × 10^−5^) to NCBI non-redundant protein (Nr) database (http://www.ncbi.nlm.nih.gov), the Swiss-Prot protein database [28], the Kyoto Encyclopedia of Genes and Genomes (KEGG) database [29], and the Clusters of Orthologous Groups database eukaryote-specific version (KOG) [30]. The best alignment results were defined as suitable protein functional annotations. The statistical analysis of annotations were showed by Venn diagrams (http://bioinformatics.psb.ugent.be/webtools/Venn/), Excel and R package ggplot2 [31]. Gene Ontology (GO) annotation was analyzed by Blast2GO [32] according to the Nr annotations and the GO functional classification of unigenes was exhibited using WEGO [33].

### 2.4. Gene Expression and Enrichment Analysis

High quality clean reads were mapped onto the total assembled unigenes using Bowtie2 [34] and then the transcript abundance of unigenes were calculated and normalized to reads per kilobase per million reads (RPKM) using RSEM [35]. The significant differentially expressed genes (DEGs) across sample groups were analysed using edgeR [36] with the threshold of a |log2Fold change| ≥ 1 and a false discovery rate (FDR) < 0.05. The statistical analysis of DEGs were exhibited by R ggplot2 [31], Venn diagrams, and Excel software. The GO enrichment analysis of DEGs was carried out using R package topGO (http://www.bioconductor.org/) with the threshold of FDR < 0.05. The KEGG enrichment analysis of DEGs was performed using KEGG Orthology Based Annotation System (KOBAS) [37] with the threshold of FDR < 0.05 and the top 20 enriched KEGG pathways were visualized using the R package ggplot2 [31].

### 2.5. Cold Regulation of Reversible Protein Phosphorylation and Ca^2+^ Signal Components 

Based on the annotation, the DEGs of protein kinases and protein phosphatases were identified and counted. Moreover, the DEGs of Ca^2+^ signal components were selected and exhibited by heatmaps using HemI [38], including calmodulin (CaM), calmodulin-like (CaML), calcineurin B-like protein (CBL), calcium-dependent protein kinase (CDPK), CBL-interacting protein kinase (CIPK), calcium/calmodulin-dependent protein kinase (CaMK), calcium/calmodulin-regulated receptor-like kinase (CRLK), and mitogen-activated protein kinase (MAPK) cascade members. All DEGs were differentially expressed in at least any one of the five stages. 

### 2.6. Cold Regulation of Major Sugars and Polysaccharides Metabolisms

The genes involved in starch and sucrose metabolism (ko00500), amino sugar and nucleotide sugar metabolism (ko00520) and Other glycan degradation (ko00511) KEGG pathway were selected according to the KEGG annotation and checked based on Nr annotation. Key enzyme genes without KEGG annotation hits were alternatively selected based on Nr annotation. These genes were mapped to the ko00500 and ko00520 pathway maps (ko00511 is no map in KEGG database) to predict the putative pathway related to major sugars and polysaccharides metabolisms in cold-stored loquat fruits. Unmapped genes were manually added to the predicted pathway based on enzyme coding (EC) numbers or literature reports. The overall expression levels of the genes associated with each enzyme were calculated based on RPKM values and showed by TBtools [39]. The predicted starch and sucrose metabolism pathway was drawn by Powerpoint software (Microsoft, Redmond, WA, USA). 

## 3. Results and Discussion

### 3.1. Transcriptome Assembly and Unigene Annotation

**Figure 1:**
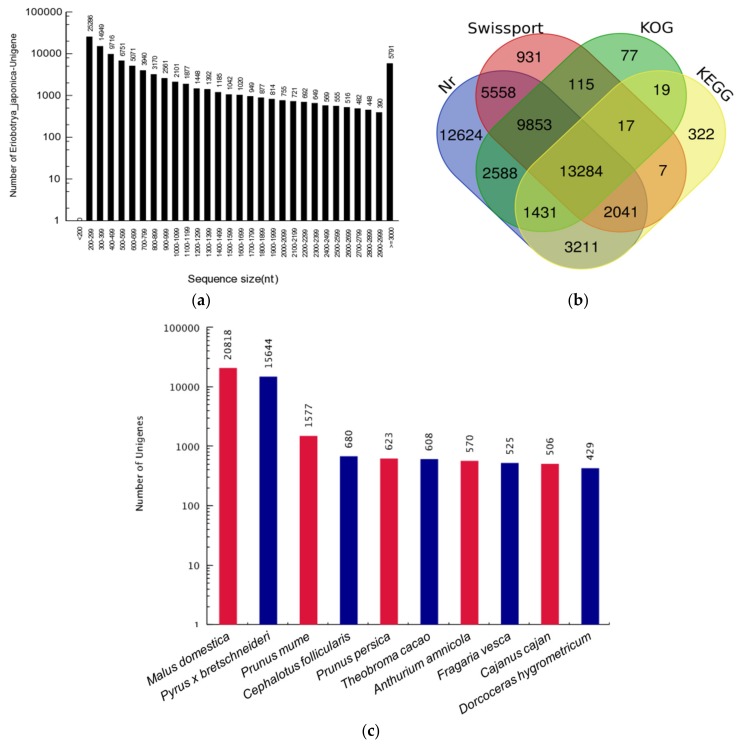

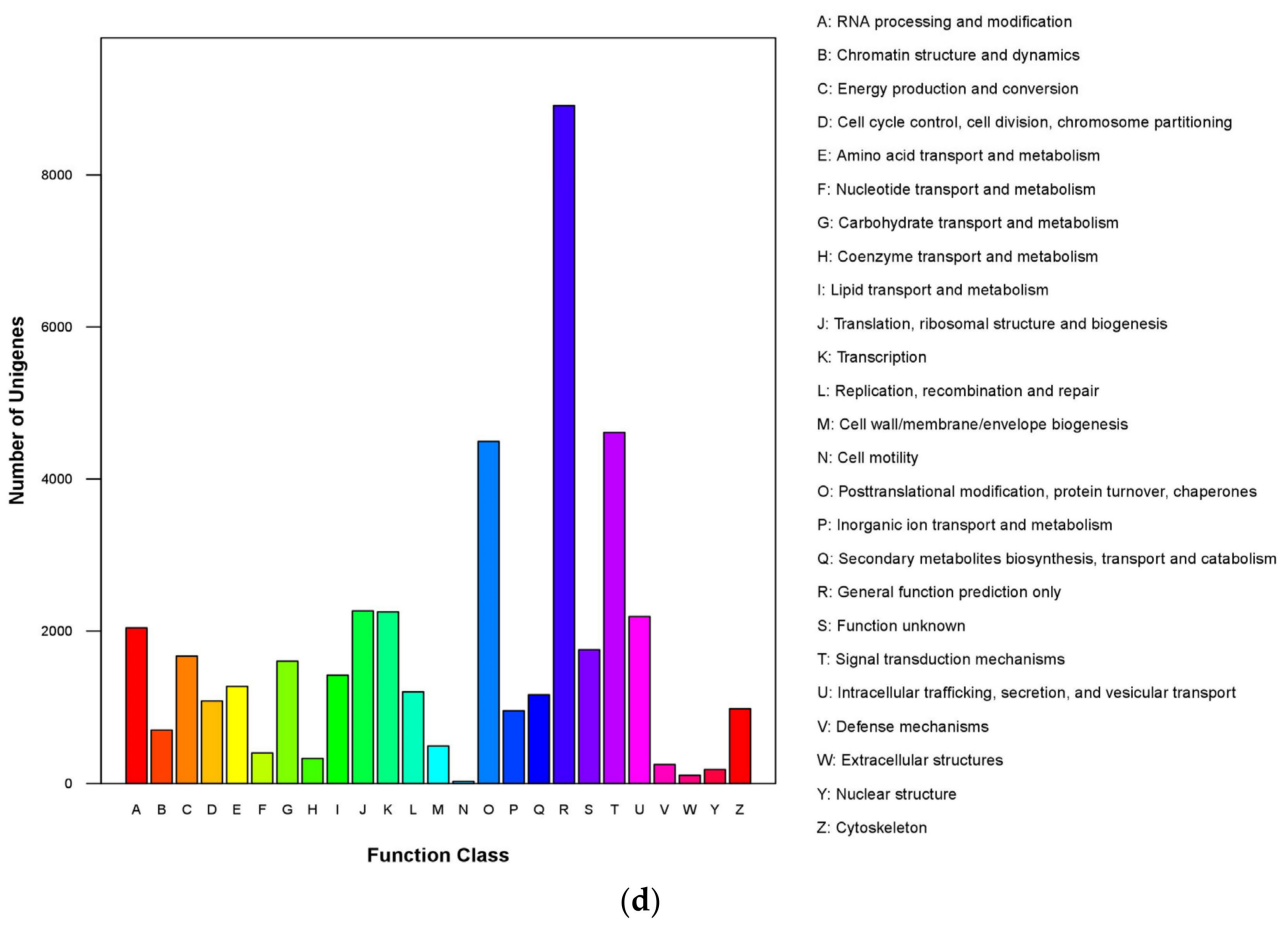
Unigene statistics and annotation. (**a**) The sequence size statistics of unigenes; (**b**) The Venn diagram of unigene annotations from Nr, Swiss-Prot, KOG (Clusters of Orthologous Groups database eukaryote-specific version), and KEGG (Kyoto Encyclopedia of Genes and Genomes) database; (**c**) The top 10 species of BLAST hits of unigenes in Nr database; (**d**) KOG function classification of unigenes.

Total RNA of cold stored loquat fruit pulps was extracted and eligible RNA samples (Figure S1) were used to cDNA library construction and Illumina transcriptome sequencing. In total, 2,289,564,890 raw reads were generated from eighteen cDNA libraries and 2,207,813,200 clean reads were obtained by filtering adapters, unknown nucleotides and low-quality reads (Table S1). The clean reads were assembled and 95,717 non-redundant unigenes were acquired with 88,349,966 bp in total length. The length of unigenes were ranged from 201 bp to 28,470 bp that the average length was 923 bp and N50 was 1724 bp. There were 89,926 unigenes (93.95%) ranging from 201 to 2999 bp and the length distribution of unigenes was inversely proportional to quantity (Figure 1a). The unigenes were annotated using BLAST against Nr, Swiss-Port, KOG, and KEGG databases and 50,589, 31,805, 27,383, and 20,311 unigenes hit annotations in these databases, respectively. There were 52,077 unigenes which had at least one hit and 13,284 unigenes had annotations in all four databases (Figure 1b). Five hundred and fifty-seven species contributed to the annotations of 50,589 unigenes in Nr database. *Malus domestica*, *Pyrus* × *bretschneideri* and *Prunus mume* were the primary donors that annotated 20,818 (41.15%), 15,644 (30.92%), and 1577 (3.12%) unigenes (Figure 1c). A total of 27,383 unigenes had KOG annotations and divided into 25 functional categories. The three categories with most unigenes were “General function prediction only”, “Signal transduction mechanisms”, and “Posttranslational modification, protein turnover, chaperones”, followed by “Transcription” and “Translation, ribosomal structure and biogenesis” (Figure 1d).

**Figure 2:**
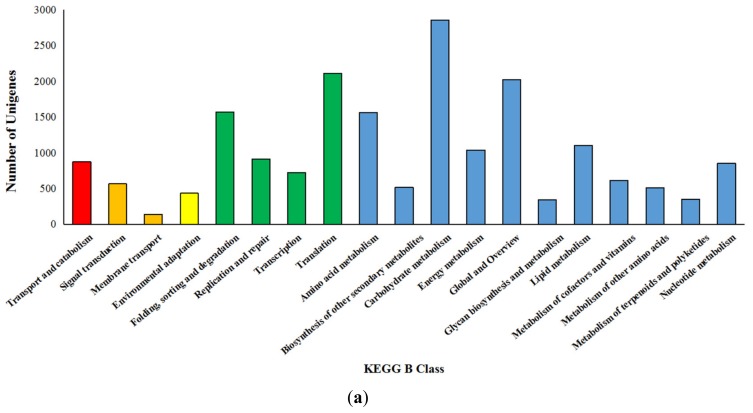

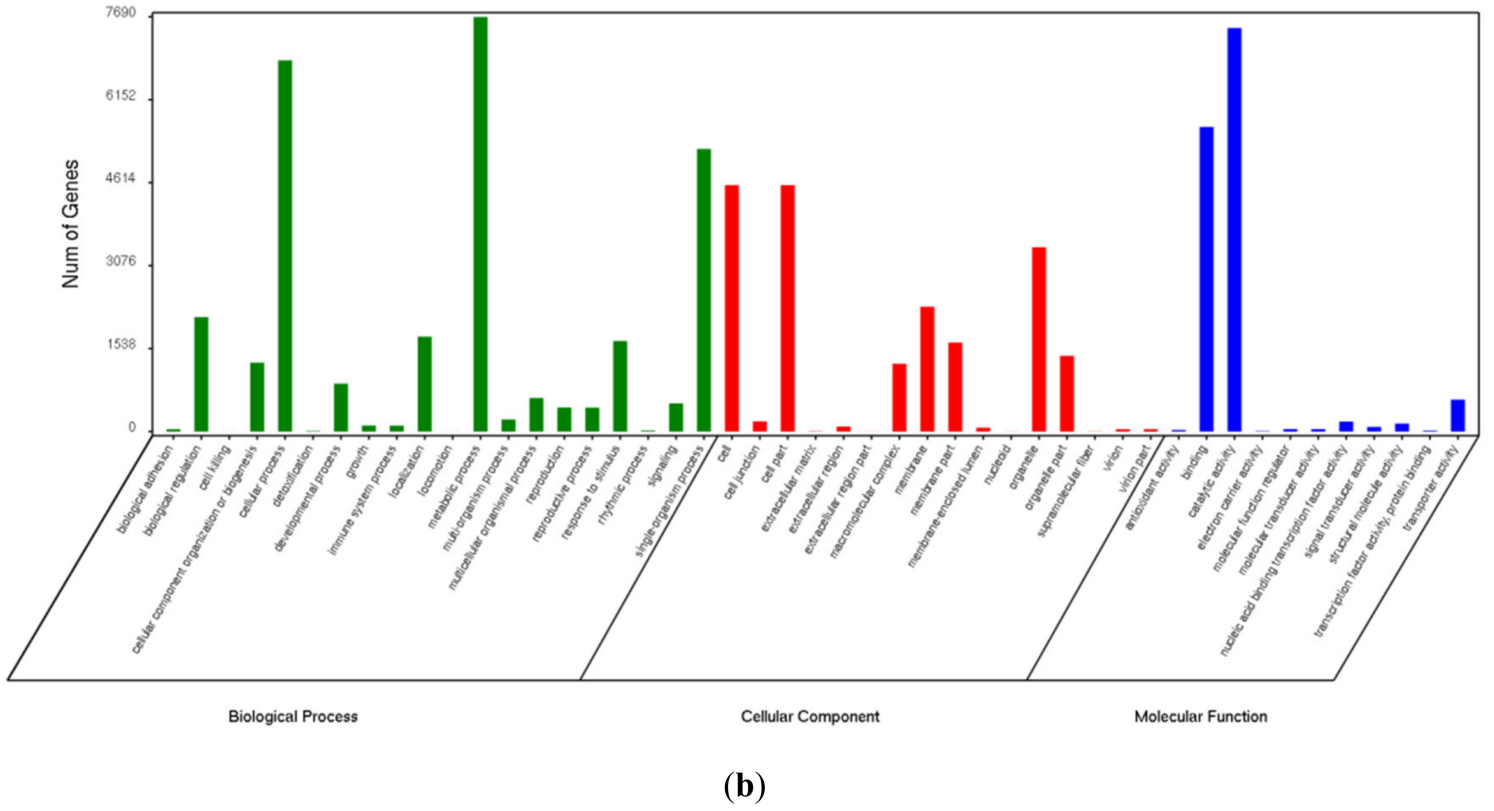
KEGG and gene ontology (GO) analysis of unigenes. (**a**) The KEGG annotation statistics of unigenes; Different colors represent KEGG A Class: Cellular Processes (red), Environmental Information Processing (orange), Organismal Systems (yellow), Genetic Information Processing (green), and Metabolism (blue); (**b**) The level 2 GO functional classification of unigenes; Different colors represent three main ontology terms: Biological Process (green), Cellular Component (red), and Molecular Function (blue).

A total of 19,094 unigenes matched 5 KEGG A Class, 19 KEGG B Class and 128 KEGG pathways. The majorly matched KEGG A Class were “Metabolism” including 11,765 (61.62%) unigenes and “Genetic Information Processing” including 5321 (27.87%) unigenes (Figure 2a). A total of 13,465 unigenes had 64,000 hits in three main ontology terms “Biological Process” (30,080 hits), “Cellular Component” (19,634 hits), and “Molecular Function” (14,286 kits). The three-most frequent-hit level 2 GO terms were “Metabolic Process”, “Cellular Process”, and “Single-organism Process” in “Biological Process”, “Cell”, “Cell Part”, and “Organelle” in “Cellular Component”, and “Binding”, “Catalytic Activity”, and “Transporter Activity” in “Molecular Function” (Figure 2b).

### 3.2. Gene Expression Analysis and Significant Differentially Expressed Genes (DEGs)

**Figure 3:**
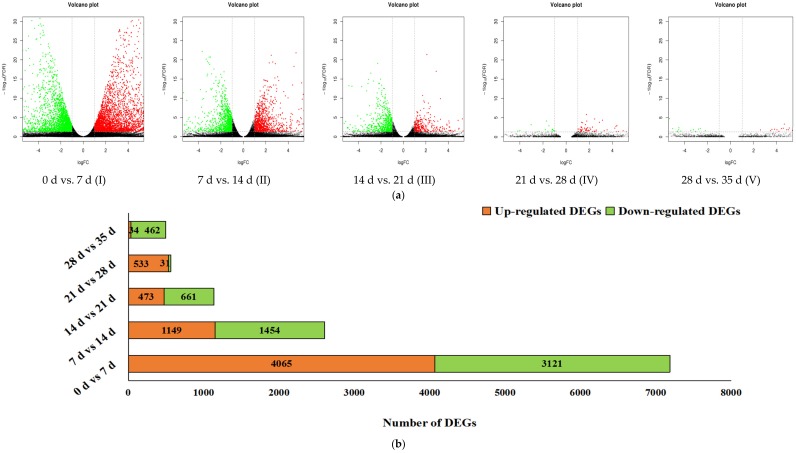

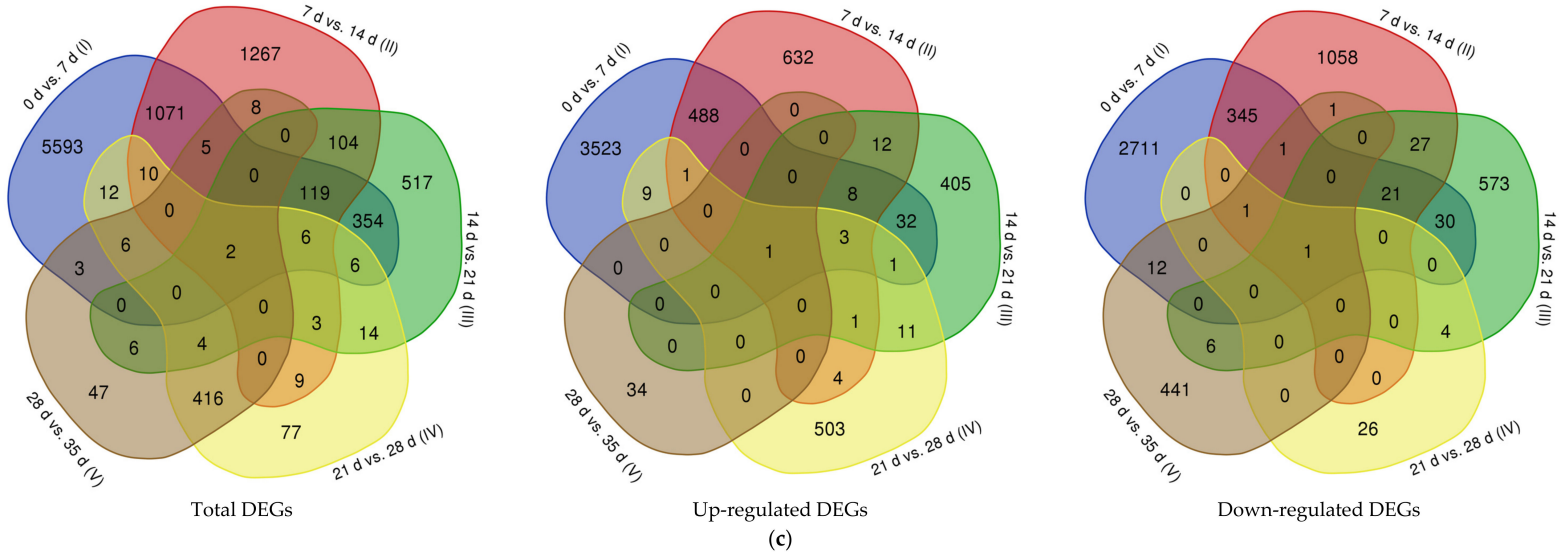
The number of differentially expressed genes (DEGs) in five stages of cold-stored loquat fruits. (**a**) Volcano plots illustrated the expression patterns of DEGs in different stages; red spots were up-regulated DEGs and green spots were down-regulated DEGs; (**b**) Barchart showed the number of DEGs detected in different stages; (**c**) Venn programs exhibited the overlapped DEGs among different stages.

Plants respond to cold by changing many physiological processes, including gene expression. A large number of cold-induced genes were induced by cold stress at the transcriptional level to encode proteins that protect against chilling injury [40]. In this work, the expression abundance of genes were calculated and the DEGs were identified by five pairwise comparisons of the six sample groups in cold-stored loquat fruits, including the stages of zero days to seven days (I), seven days to 14 days (II), 14 days to 21 days (III), 21 days to 28 days (IV), and 28 days to 35 days (V). A total of 7186 DEGs were detected in the first seven d and sharply decreased to 2603 from seven d to 14 d and 1134 from 14 d to 21 d. After that, DEGs were sequentially reduced to 564 and 496 from 21 d to 35 d (Figure 3a,b). Obviously, the cold-induced DEGs were intensely expressed in the earlier stage, which helped loquat fruits to adjust their metabolisms and tolerate chilling injury. In contrast, the quickly decreasing DEGs in later stages implied the loquat fruits was gradually adapting to cold storage environment. Moreover, a total of 5593, 1267, 517, 77, and 47 genes were specific DEGs in the five stages of I, II, III, IV, and V, respectively. A total of 1997 DEGs were differentially expressed both in two stages and a few DEGs were differentially expressed in more than three stages. No common genes were invariably found to be up-regulated or down-regulated during cold storage (Figure 3c). It was suggested that the expression patterns were differed among the five stages. Previous researches of cold-stored loquat fruits have displayed the changes of metabolites and enzyme activities [5,6,7,8,9,10]. The synthesis of lignin in cold-stored loquat fruits has been clearly understood that is the results of the coordinated action of many phenylpropanoid biosynthesis related enzymes, which were regulated by the expression of encoding genes and transcriptional factors [1,6,11,12,13,14,15,16,17,18]. Therefore, the relationships between gene expression and metabolite accumulation need to be further explored.

### 3.3. Gene Ontology Enrichment Analysis of Differentially Expressed Genes

Gene ontology classification and functional enrichment were performed for the DEGs of five stages. A total of 24 (2096 Hits), seven (344 Hits), 12 (196 Hits), eight (277 Hits), and 88 (449 Hits) Go terms were enriched in the stage of zero d to seven d (I), seven d to 14 d (II), 14 d to 21 d (III), 21 d to 28 d (IV), and 28 d to 35 d (V), respectively (Table 1). In Cellular Component terms, the enrichment GO terms were five membrane-related (GO:0016020, GO:0030312, GO:0031224, GO:0044425, GO:0071944) GO terms both in stage I and II; three membrane-related (GO:0016021, GO:0031224, GO:0044425) and three plastid-related GO terms (GO:0009507, GO:0044434, GO:0044435) in stage III; four cell-related (GO:0044464, GO:0005576, GO:0005622, GO:0005623), two cytoplasm-related (GO:0005737, GO:0044444) and one plastid-related (GO:0009536) GO terms in stage IV; four cell-related (GO:0005622, GO:0005623, GO:0044424, GO:0044464), two cytoplasm-related (GO:0005737, GO:0044444), one plastid-related (GO:0009536) and four organelle-related (GO:0043226, GO:0043227, GO:0043229, GO:0043231) GO terms in stage V (Table S2). Obviously, the enriched DEGs were located from membrane to intracellular cytoplasm and organelles as storage time goes by, which indicated that membrane-related genes preferentially responded to low temperature and followed by intracellular protein genes. 

In Molecular Function, the enrichment GO terms were six oxidoreductase activity-related (GO:0016491, GO:0016639, GO:0016679, GO:0016682, GO:0016705, GO:0052592), three reversible protein phosphorylation-related (GO:0004672, GO:0016301, GO:0016773), three glycosyl hydrolase activity-related (GO:0004553, GO:0015926, GO:0016798), four binding-related (GO:0005506, GO:0032553, GO:0043169, GO:0046906), one transmembrane transporter activity-related (GO:0015291) and glutamate synthase activity (GO:0045181) GO terms in stage I; two oxidoreductase activity-related (GO:0016706, GO:0051213) GO terms in stage II; four oxidoreductase activity-related (GO:0016491, GO:0016651, GO:0016679, GO:0016682) and two binding-related (GO:0046906, GO:0048037) GO terms in stage III; oxidoreductase activity (GO:0016491) GO terms in stage IV; two oxidoreductase activity-related (GO:0016491, GO:0000104) GO terms in stage V (Table S3). In Biological Process, the enrichment GO terms were polyamine metabolic process (GO:0006595) GO terms in stage I and two small molecule metabolic-related and four organic acid metabolic-related GO terms in stage V. There were no enriched GO terms in stage II, III, and IV (Table S4). The results suggested that the DEGs in response to cold storage environment were more complex in earlier stage, including oxidoreductase, reversible protein phosphorylation, glycosyl hydrolase, binding, transmembrane transporter, glutamate synthase, and polyamine metabolism related genes. While in later stage, the DEGs were mainly enriched in oxidoreductase activity related GO terms, indicating loquat fruits had adapted to the cold storage environment.

### 3.4. Kyoto Encyclopedia of Genes and Genomes Enrichment Analysis of Differentially Expressed Genes 

**Figure 4:**
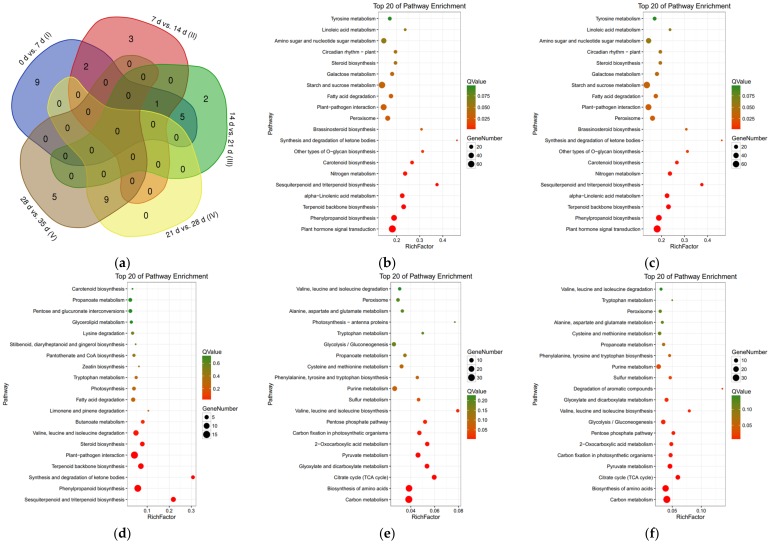
The KEGG enrichment analysis of DEGs in five stages of cold-stored loquat fruits. (**a**) Venn programs showed the overlapped enriched KEGG pathways among different stages; (**b**) 0 days vs. 7 days (I); (**c**) 7 days vs. 14 days (II); (**d**) 14 days vs. 21 days (III); (**e**), 21 days vs. 28 days (IV); (**f**) 28 days vs. 35 days (V).

To further understand the related pathways of DEGs, KEGG enrichment analysis was carried out for the DEGs of five stages. A total of 17 (1085 DEGs), six (351 DEGs), eight (143 DEGs), nine (145 DEGs), and 14 (129 DEGs) pathways were enriched in the stage of zero days to seven days (I), seven days to 14 days (II), 14 days to 21 days (III), 21 days to 28 days (IV), and 28 days to 35 days (V), respectively (Table S5). There were no intersections among four or five stages. Only phenylpropanoid biosynthesis (ko00940) was significantly enriched in the earlier three stages. Starch and sucrose metabolism (ko00500) and carotenoid biosynthesis (ko00906) were both enriched in stages I and II. Five pathways including steroid biosynthesis (ko00100), plant–pathogen interaction (ko04626), sesquiterpenoid and triterpenoid biosynthesis (ko00909), synthesis and degradation of ketone bodies (ko00072), terpenoid backbone biosynthesis (ko00900) were both enriched in stages I and III. Nine pathways including carbon fixation in photosynthetic organisms (ko00710), pentose phosphate pathway (ko00030), pyruvate metabolism (ko00620), valine, leucine, and isoleucine biosynthesis (ko00290), glyoxylate and dicarboxylate metabolism (ko00630), citrate cycle (TCA cycle) (ko00020), 2-oxocarboxylic acid metabolism (ko01210), carbon metabolism (ko01200), and biosynthesis of amino acids (ko01230) were both enriched in stage IV and V (Figure 4). For stage-specific pathways, nine pathways brassinosteroid biosynthesis (ko00905), nitrogen metabolism (ko00910), other types of O-glycan biosynthesis (ko00514), plant hormone signal transduction (ko04075), fatty acid degradation (ko00071), circadian rhythm-plant (ko04712), peroxisome (ko04146), galactose metabolism (ko00052), and alpha-linolenic acid metabolism (ko00592) were only enriched in stage I (Figure 4b); three pathways including pentose and glucuronate interconversions (ko00040), limonene and pinene degradation (ko00903), amino sugar and nucleotide sugar metabolism (ko00520) in stage II (Figure 4c); two pathways including valine, leucine and isoleucine degradation (ko00280), butanoate metabolism (ko00650) in stage III (Figure 4d); five pathways including sulfur metabolism (ko00920), glycolysis/gluconeogenesis (ko00010), 2-oxocarboxylic acid metabolism (ko01220), purine metabolism (ko00230), phenylalanine, tyrosine and tryptophan biosynthesis (ko00400) in stage V (Figure 4f). There was no pathway specifically enriched in stage IV (Figure 4e). The results indicated that cold-induced pathways were mainly concerned with signal transduction and secondary metabolism (lignin, terpenoid, carotenoid, steroid) in the first three stages and were majorly related to primary metabolism in the later two stages, especially energy metabolism.

### 3.5. Cold Regulation of Reversible Protein Phosphorylation

Gene Ortology enrichment results suggested that many DEGs were enriched in the GO terms related to reversible protein phosphorylation. Based on the annotation, the DEGs involved in reversible protein phosphorylation were selected and counted. A total of 503 transcripts of protein kinases and 106 transcripts of protein phosphatases were differentially expressed in at least any one of the five stages. DEGs of protein kinases were more than protein phosphatases. In earlier stages, DEGs of reversible protein kinases and phosphatases were both more than later stages (Table 2). Interestingly, up-regulated DEGs of protein kinases were significantly more than down-regulated DEGs of that, while DEGs of protein phosphatase were in reverse (Table 2). The results showed that reversible protein phosphorylation related DEGs were significant for cold response in loquat fruits, especially in earlier stages. The products of gene expression functioned not only in cold tolerance but also in the regulation of gene expression and signal transduction in cold responses [41]. Signal transduction pathways connected the cold sensing mechanism and the down-stream genetic response [42]. Kinases and phosphatases regulated reversible phosphorylation can either activate or inactivate enzyme activity and thus controlling the most diverse biological pathways including signaling [43].

### 3.6. Cold Regulation of Ca^2+^ Signal Components

**Figure 5:**
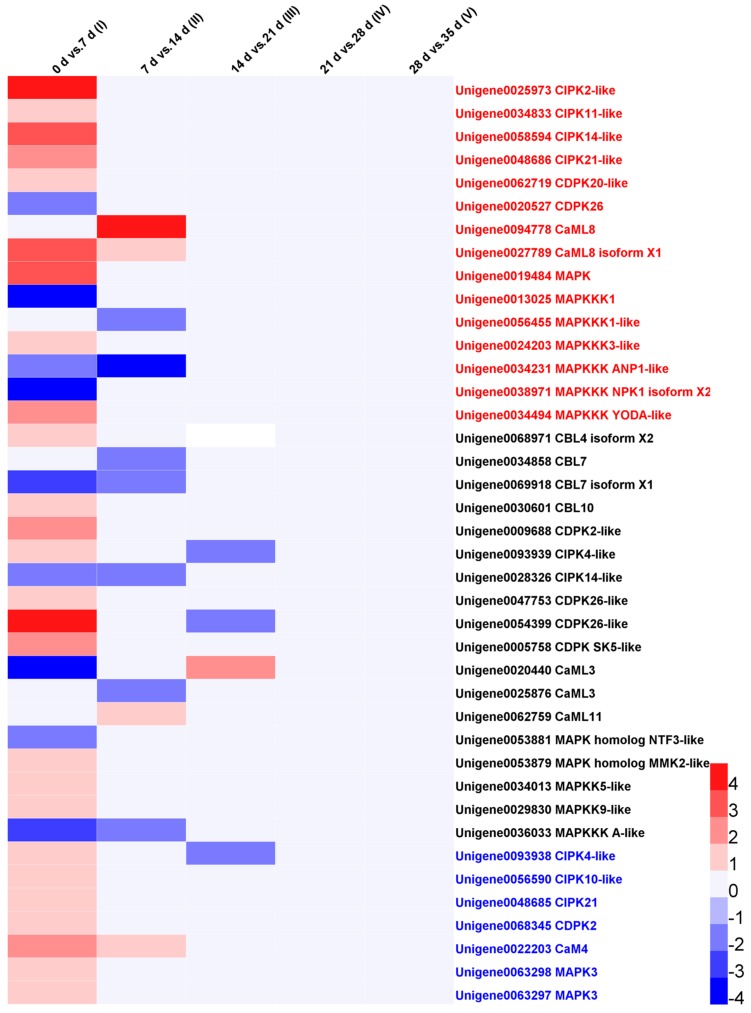
The heatmap showed the DEGs expression abundance of Ca^2+^ signal components in five stages of cold-stored loquat fruits. Different colors represents the log_2_ (fold change) value of the DEGs in the stages. Different right-label colors represents the reads per kilobase per million reads (RPKM) value of the DEGs. Red: low expression abundance genes (RPKM < 5); black: moderate expression abundance genes (5 ≤ RPKM < 50); blue: high expression abundance genes (RPKM ≥ 5).

The cytosolic Ca^2+^ is considered as a crucial second messenger in cold signal transduction and cold acclimation development. Nuclear Ca^2+^ signaling is also essential in transcriptional regulation especially the expression of cold-induced genes [44]. The cold-induced Ca^2+^ signature can be sensed by different sensors and downstream protein kinases which transduced the cold signal to switch on gene transcription, including CaM/CaML, CBL, CDPK, CIPK, CaMK, CRLK and MAPK cascade members [42,44,45,46,47]. We screened the Ca^2+^ signal components which were differentially expressed in at least any one of the five stages. As shown in Figure 5, there were 40 DEGs that were related to Ca^2+^ signal pathways including one CaM, five CaML, four CBL, seven CDPK, nine CIPK, and 14 MAPK cascade members (five MAPK, two MAPKK, and seven MAPKKK). These DEGs showed dynamic expression trends in earlier three stages and there were no DEGs involved in two later stages. Twenty-six DEGs were up-regulated and nine were down-regulated in stage I; four DEGs were up-regulated and seven were down-regulated in stage II; Only one DEGs were up-regulated and three were down-regulated in stage III; 15, 18, and seven DEGs were low, moderate, and high expression abundance genes, respectively. All the seven high-expressed DEGs were up-regulated in the first stage and two of them were which were down-regulated in one later stage (Figure 5.). The results suggested the important signaling functions of CDPK, CBL-CIPK, and CaM/CaML-MAPK cascade in cold-stored loquat fruits.

### 3.7. Cold Regulation of Major Sugars and Polysaccharides Metabolisms

**Figure 6:**
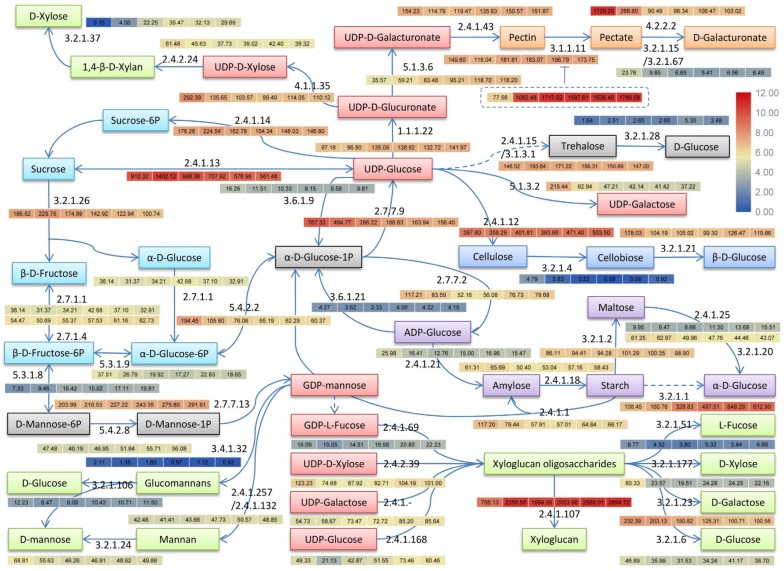
The predicted pathway of major sugars and polysaccharides metabolisms in cold-stored loquat fruits. Red boxes: NDP-sugars; sky blue boxes: sucrose metabolism; purple boxes: starch metabolism; blue boxes: cellulose metabolism; orange boxes: pectin metabolism; green boxes: hemicelluloses metabolisms; gray boxes: other metabolites; dashed box: pectin methylesterase inhibitor. Solid arrow represents the reaction is direct while dashed arrow means there are several reactions between these two metabolites. The numbers next to arrows (i.e., 3.2.1.26) are enzyme codes (Table 3). Dispersive heatmaps with different colors next to enzyme codes exhibited the log_2_ reads per kilobase per million reads (RPKM) value of the enzymes and the numbers in different boxes exhibit the corresponding RPKM values. The heatmap boxes from left to right were zero days, seven days, 14 days, 21 days, 28 days, and 35 days, respectively.

According to the KEGG and Nr annotation, there were 49 enzymes encoded by 649 genes that related to major sugars and polysaccharides metabolisms in cold-stored loquat fruits (Table 3). Most enzymes had multiple coding genes, which indicated that most reactions might be catalyzed by some isozymes encoded by different genes. Based on these genes, a putative pathway were predicted and the gene expression of each enzymes were also quantified through RPKM value (Figure 6). The predicted pathway exhibited the metabolism of major sugars and polysaccharides including sucrose, fructose, glucose, starch, pectin, cellulose, and hemicelluloses. Our results provided the transcriptomic evidence to further understand the changes of these saccharides in cold-stored loquat fruit. 

#### 3.7.1. Major Sugars and Starch Metabolisms

Glucose, fructose, and sucrose are the dominant sugars in postharvest loquat fruits [9]. We found that the expression trend of enzyme encoding genes involved in sucrose, fructose and glucose transformation (EC:2.4.1.13, EC:2.4.1.14, EC:3.2.1.26, EC:5.3.1.9, EC:5.4.2.2, EC:2.7.7.9, EC:3.6.1.9) were generally down-regulated, indicating the sucrose, fructose, and glucose transformation was restained by cold storage. It has been reported that the activity changes of sucrose synthase (EC:2.4.1.13), sucrose-phosphate synthase (EC:2.4.1.14) and invertase (EC:3.2.1.26) lead to the steady decrease of sucrose in cold-stored loquat fruits. Especially, sucrose synthase principally performed the increasing cleavage activity which split sucrose into fructose and UDP-glucose [9,10]. However, the expression of these three enzyme genes showed a similar expression pattern in this work, suggesting the existence of further posttranscriptional regulations. Despite of that, the residual high abundance of them might still contribute to the number of corresponding enzymes. In contrast, the stable expression of hexokinase (EC:2.7.1.1) and fructokinase (EC:2.7.1.4) in our research implied the sustaining phosphorylation of fructose and glucose, which subsequently entered into glycolysis and TCA cycle to consume for energy metabolism. A similar consumption mechanism of soluble sugars was also reported in cold-stored orange fruits [48]. The encoding genes of three starch synthesis related enzymes (EC:2.7.7.27, EC:2.4.1.21, EC:2.4.1.8) were firstly down-regulated and then up-regulated. The encoding genes of three enzymes (EC:3.2.1.1, EC3.2.1.2, EC:2.4.1.1) that catalyzed three starch hydrolysis pathways showed different expression trends. Among them, the expression abundance of α-amylase (EC:3.2.1.1) encoding genes were higher than others and significantly up-regulated. It suggested that cold storage suppressed the synthesis of starch and accelerated the starch hydrolysis to form α-D-Glucose mainly through α-amylase catalyzed pathway. It has been described that glucose and fructose contents first increased and then decreased in chilling injury loquat fruits [9,10]. These results indicated that the hydrolysis of starch and sucrose both contributed to the accumulation of glucose and fructose in the earlier stages, while the decrease contents of glucose and fructose might be due to the exhausted starch and sucrose and active energy metabolism. 

Our results also showed the expression of enzyme genes related to the synthesis of trehalose. Trehalose is a nonreducing sugar that functions as stress protector in a variety of organisms [49]. The expression abundance of trehalose 6-phosphate synthase/phosphatase (TPS/TPP) (EC:2.4.1.15/3.1.3.12) genes were much higher than α,α-trehalase (EC:3.2.1.28) genes, which might contribute to the accumulation of trehalose. In rice, an OsMAPK3-OsICE1-OsTPP1 signal cascade has been reported that induced the producing of trehalose to give rice cold tolerance [45]. The expression of loquat MAPK3 was also cold-induced in our research. Thus, the MAPK3-ICE1-TPP1 signaling transduction and trehalose accumulation might be the cold responses in loquat fruits.

#### 3.7.2. Cell Wall Polysaccharides Metabolisms

The nucleotide sugars (NDP-sugars) in the pathway can also be used as precursors for the synthesis of cell wall polysaccharides. The activated and various NDP-sugars were catalyzed by different glycosyltransferases (GTs, 2.4.) to form growing polysaccharide chains [50]. The cell wall polysaccharides changes in cold-stored loquat fruits exhibited the decreasing water-soluble pectin content and increasing protopectin, hemicellulose and cellulose [5,51]. Cellulose is linear β-1,4-glucan polymer chains that extended by the catalyzing of cellulose synthases at the plasma membrane from precursor UDP-glucose. These chains spontaneously cocrystallize into microfibrils which are inelastic and thought to contribute to rigidity of cell walls [52]. We found that cellulose synthase (EC:2.4.1.12) genes were highly and stably expressed while the expression abundance of endoglucanase/cellulase (EC:3.2.1.4) genes were low and greatly limited by cold storage, which lead to the accumulation of cellulose.

Pectins contribute strength and flexibility to the cell wall [52]. Pectin-rich cell walls play a key role in cell–cell and/or tissue cohesion interfaces in middle lamella and cellular junctions [5]. Homogalacturonan (HG) is the most abundant cell wall pectin with a 70–80% methyl-esterified form [53]. Pectin methylesterase (PME) catalyze the removal of methyl esters to enhance the susceptibility of HG hydrolysis that was catalyzed by polygalacturonase (PG) and pectate lyase (PL) within the wall [54]. The PME and PG activities were both repressed that contributed to the pectin changes in cold-stored loquat fruits [5]. In our results, the expression abundance of UDP-glucuronate 4-epimerase (EC:5.1.3.6) genes were obviously increased that might stimulate the active of pectin synthesis pathway. The α-1,4-galacturonosyltransferase (EC:2.4.1.43) and PME (EC:3.1.1.11) genes firstly down-regulated and then up-regulated with the higher expression abundance than UDP-glucuronate 4-epimerase (EC:5.1.3.6), which participated in the synthesis and hydrolysis of pectin, respectively. However, an interesting finding was that the PME inhibitor genes were sharply up-regulated in the earlier stages and then maintained high level. PME activity could be restricted by PME inhibitors (PMEI) [55]. The accumulation of PMEI transcripts were also observed in cold-stored peach [24]. Therefore, it was implied that the activity of PME was strongly repressed by PME inhibitors in cold-stored loquat fruits, thereby suppressing the removal of pectin methyl esters. Besides, the expression abundance of endo-/exo-polygalacturonase (endo-/exo-PG) (EC:3.2.1.15/EC:3.2.1.67) and PL (EC:4.2.2.2) genes were obviously decreased in cold-stored loquat fruits, which inhibited the degradation of pectate. 

Hemicelluloses are polysaccharides other than cellulose or pectins, which mainly contained xylans, xyloglucans, glucomannans and mannans. Hemicelluloses are synthesized by glycosyltransferases and interacted with cellulose microfibrils to strengthen the cell wall [56]. Xylans are the major component of hemicellulose in the secondary cell walls of dicotyledonous plants. Xylan is composed of a backbone of β(1,4)-linked xylose chain and may contain some side branches such as arabinose, glucuronic acid and 4-O-methyl glucuronic acid [57]. Our result displayed that UDP-glucuronate decarboxylase (EC:4.1.1.35) genes were remarkably down-regulated to reduce the synthesis of xylan precursor UDP-D-Xylose. The 1,4-β-d-xylan synthase (EC:2.4.2.24) genes were slightly down-regulated and β-d-xylosidase (EC:3.2.1.37) genes were rapidly up-regulated. Despite of these, the expression abundance of 1,4-β-d-xylan synthase genes was still higher than β-d-xylosidase genes, which might lead to the accumulation of xylans.

Xyloglucans are the most abundant hemicellulose in the primary cell walls of dicotyledonous plants [54]. Xyloglucan is made up of a backbone of β(1,4)-linked glucose residues and generally branched with α(1,6)-linked xylose residues which often link to a β(1,2)-linked galactose residue sometimes followed by a α(1,2)-linked L-fucose residue. There were 9 enzymes participate in the metabolisms of xyloglucan [58]. The xyloglucan often contains glucose, xylose, and galactose in a molar ratio of approximately 4:3:1, which function as a storage polysaccharide. In contrast, the fucosylated xyloglucan is a structure polysaccharide in cell wall, which contain glucose, xylose, galactose and fucose of approximately 4:3:1:1 [59]. Our results displayed that xyloglucan glycosyltransferase (EC:2.4.1.168), xyloglucan 6-xylosyltransferase (EC:2.4.2.39), galactoside 2-α-l-fucosyltransferase (EC:2.4.1.69) genes were down-regulated in the first stages and then up-regulated and xyloglucan galactosyltransferase (EC:2.4.1.?) genes were gradually up-regulated, indicating that the synthesis of xyloglucan was enhanced. Besides, Endo-1,3;1,4-β-d-glucanase (EC:3.2.1.6), α-xylosidase (EC:3.2.1.177), α-l-fucosidase (EC:3.2.1.51), and β-galactosidase (EC:3.2.1.23) were down-regulated, suggesting that the degradation of xyloglucan was weakened. For glucose, xylose, and fucose residues of xyloglucan, the expression abundance of synthesis-related enzyme genes were higher than degradation-related enzyme genes. For galactose residues of xyloglucan, the expression abundance of synthesis-related enzyme genes were far less than degradation-related enzyme genes in the first stage and became close in the latest two stage. These gene expression patterns implied the accumulation of xyloglucans in cold-stored loquat fruits, especially the structure xyloglucans. Xyloglucan endotransglucosylase/hydrolase (EC:2.4.1.207) catalyzes the reversible formation of xyloglucan and grafts new xyloglucan molecules into the cell wall structure [60]. The enzyme genes were obviously up-regulated with the highest expression abundance among all enzyme genes in the predicted pathway, which illustrated the active cell wall strengthening in cold-stored loquat fruits.

Mannans have structural functions that cross-link cellulose and other main hemicelluloses in cell walls [61]. The expression abundance of α-1,3/1,6-mannosyltransferase (EC:2.4.1.257/2.4.1.132) genes were similar to α-mannosidase (EC:3.2.1.24) genes, indicating the content of mannans was relatively stable. Glucomannan is a structural hemicellulose in plant secondary cell walls, which is consist of β(1,4)-linked D-mannose and D-glucose at a ratio of 1.6:1, with about 8% branching [62]. The expression abundance of glucomannan 4-β-mannosyltransferase (EC:3.4.1.32) were far lower than mannosyl-oligosaccharide glucosidase (EC:3.2.1.106) that suggested the glucomannan might be degraded. Therefore, the increased content of hemicellulose in cold-stored loquat fruits might attribute to the accumulations of xylans and xyloglucans, which were caused by the expression of related enzyme genes.

## 4. Conclusions

In conclusion, we reported the transcriptomic responses in *E. japonica* fruits during postharvest cold storage using the RNA-seq technology and de novo analysis based on Illumina HiSeq^TM^ 4000 platform. Firstly, our results displayed the overall transcriptomic responses of loquat fruits under postharvest cold storage stress. Besides, we found that protein kinases and phosphatases, and Ca^2+^ signal components were related to the cold adaption of postharvest loquat fruits. Finally, we predicted a putative pathway related to the major sugars and polysaccharides metabolisms to further investigate the changes of these saccharides in cold-stored loquat fruit. Taken together, these results provide a foundation and orientation for future studies on improving the postharvest life of *E. japonica*.

## Figures and Tables

**Table 1:** Gene ontology (GO) terms enrichment statistics.

GO Term	0 d vs. 7 d (I)		7 d vs. 14 d (II)		14 d vs. 21 d (III)		21 d vs. 28 d (IV)		28 d vs. 35 d (V)	
	Num.	Hits	Num.	Hits	Num.	Hits	Num.	Hits	Num.	Hits
Cellular Component	5	973	5	332	6	125	7	253	11	420
Molecular Function	18	1114	2	12	6	73	1	24	2	23
Biological Process	1	9	0	0	0	0	0	0	75	6
Total	24	2096	7	344	12	198	8	277	88	449

**Table 2:** DEGs number involved in protein phosphorylation of cold-stored loquat fruits.

Category	0 d vs. 7 d (I)		7 d vs. 14 d (II)		14 d vs. 21 d (III)		21 d vs. 28 d (IV)		28 d vs. 35 d (V)	
	Up	Down	Up	Down	Up	Down	Up	Down	Up	Down
Protein Kinase	282	130	103	36	31	21	10	0	1	10
Protein Phosphatase	35	50	9	16	5	2	3	0	0	2

**Table 3:** Statistics of genes involved in starch and sucrose metabolism (ko00500) pathway.

EC 1 ID	Description	KO 2 ID	Gene Number
EC:1.1.1.22	UDP-glucose 6-dehydrogenase	K00012	6
EC:2.4.1.?	Xyloglucan galactosyltransferase	Nr 3	19
EC:2.4.1.1	Starch phosphorylase	K00688	12
EC:2.4.1.12	Cellulose synthase	Nr	42
EC:2.4.1.13	Sucrose synthase	K00695	21
EC:2.4.1.14	Sucrose-phosphate synthase	K00696	10
EC:2.4.1.15/3.1.3.12	Trehalose 6-phosphate synthase/phosphatase	K16055/K01087	29
EC:2.4.1.18	1,4-α-glucan branching enzyme	K00700	15
EC:2.4.1.21	Starch synthase	K00703	7
EC:2.4.2.24	1,4-β-d-xylan synthase	Nr	6
EC:2.4.1.25	4-α-glucanotransferase	K00705	5
EC:2.4.1.257/2.4.1.132	α-1,3/1,6-mannosyltransferase	Nr	6
EC:2.4.1.43	α-1,4-galacturonosyltransferase	K13648	18
EC:2.4.1.69	galactoside 2-α-l-fucosyltransferase	Nr	3
EC:2.4.1.168	Xyloglucan glycosyltransferase	Nr	7
EC:2.4.1.207	Xyloglucan endotransglucosylase/hydrolase	Nr	20
EC:2.4.2.39	Xyloglucan 6-xylosyltransferase	Nr	3
EC:2.7.1.1	Hexokinase	K00844	10
EC:2.7.1.4	Fructokinase	K00847	11
EC:2.7.7.13	Mannose-1-phosphate guanylyltransferase	K00966	13
EC:2.7.7.27	Glucose-1-phosphate adenylyltransferase	K00975	18
EC:2.7.7.9	UTP-glucose-1-phosphate uridylyltransferase	K00963	6
EC:3.1.1.11	Pectin methylesterase	K01051	45
EC:3.2.1.1	α-amylase	K01176	16
EC:3.2.1.15/3.2.1.67	Endo-/Exo-polygalacturonase	K01184/K01213	15
EC:3.2.1.2	β-amylase	K01177	49
EC:3.2.1.4	Endoglucanase/cellulase	K01179/K19356	6
EC:3.2.1.6	Endo-1,3;1,4-β-d-glucanase	Nr	14
EC:3.2.1.20	α-glucosidase	K01187	6
EC:3.2.1.21	β-glucosidase	K01188/K05349	68
EC:3.2.1.23	β-galactosidase	K12309	8
EC:3.2.1.24	α-mannosidase	K01191	5
EC:3.2.1.26	Invertase	K01193	7
EC:3.2.1.28	α,α-trehalase	K01194	5
EC:3.2.1.37	β-d-xylosidase	K15920	3
EC:3.2.1.51	α-l-fucosidase	K01206	8
EC:3.2.1.106	Mannosyl-oligosaccharide glucosidase	Nr	9
EC:3.2.1.177	α-xylosidase	Nr	3
EC:3.4.1.32	Glucomannan 4-β-mannosyltransferase	Nr	6
EC:3.6.1.9	Ectonucleotide pyrophosphatase	K01513	2
EC:3.6.1.21	ADP-sugar diphosphatase	K18447	1
EC:4.1.1.35	UDP-glucuronate decarboxylase	K08678	14
EC:4.2.2.2	Pectate lyase	Nr	26
EC:5.1.3.2	UDP-glucose 4-epimerase	K01784	7
EC:5.1.3.6	UDP-glucuronate 4-epimerase	K08679	8
EC:5.3.1.8	Mannose-6-phosphate isomerase	K01809	7
EC:5.3.1.9	Glucose-6-phosphate isomerase	K01810	12
EC:5.4.2.2	Phosphoglucomutase	K01835	8
EC:5.4.2.8	Phosphomannomutase	K17497	4
Total	49		649

^1^ EC: Enzyme code; ^2^ KO: KEGG Orthology;^3^ Nr: this enzyme was screened by Nr annotation.

## Supplementary Materials

Supplementary materials can be found at http://www.mdpi.com/2073-4425/9/12/639/s1. Figure S1: The quality of RNA samples, Table S1: Sequencing data statistics, Table S2: GO enrichment analysis of DEGs in Cellular Component, Table S3: GO enrichment analysis of DEGs in Molecular Function, Table S4: GO enrichment analysis of DEGs in Biological Process, Table S5: KEGG enrichment analysis of DEGs.
