# Supplementary material for: De Novo Analysis Reveals Transcriptomic Responses in Eriobotrya japonica Fruits during Postharvest Cold Storage
Source: Genes (Basel). 2018 Dec 17;9(12):639. doi: 10.3390/genes9120639 (PMC6316545; doi:10.3390/genes9120639)
Supplement: Supplementary file 1 [file genes-09-00639-s001.pdf]

genes MDPI

Supplementary Materials

Table S1. Sequencing data statistics.

Sample	Raw Reads	Clean Reads (%)	GC	Clean Data (nt)	Q20 (%)	Q30 (%)
J00M-1	149590892	144811450 (96.8%)	48.13%	20862967492	20179016146 (96.72%)	18945722227 (90.81%)
J00M-2	126080430	121727188 (96.55%)	46.52%	17543618086	16968721135 (96.72%)	15935525849 (90.83%)
J00M-3	113205034	109667224 (96.87%)	46.43%	16111894522	15749293635 (97.75%)	15072712341 (93.55%)
J01M-1	119683630	115863224 (96.81%)	46.43%	16646832586	16110148996 (96.78%)	15112440002 (90.78%)
J01M-2	123911050	118791508 (95.87%)	46.51%	16971145878	16367630961 (96.44%)	15285957780 (90.07%)
J01M-3	142448062	138193074 (97.01%)	47.43%	19855343789	19213121904 (96.77%)	18025034343 (90.78%)
J02M-1	121844734	115453510 (94.75%)	46.14%	16833436142	16438980310 (97.66%)	15760324261 (93.63%)
J02M-2	124862008	120583974 (96.57%)	46.56%	17705344866	17370765233 (98.11%)	16755127175 (94.63%)
J02M-3	123672616	119407688 (96.55%)	46.89%	17532827498	17201697590 (98.11%)	16589459009 (94.62%)
J03M-1	128648556	123738840 (96.18%)	46.31%	18116830440	17750809588 (97.98%)	17091023991 (94.34%)
J03M-2	127647894	124416522 (97.47%)	46.54%	18308570345	17960444198 (98.10%)	17302510211 (94.50%)
J03M-3	124153748	121180036 (97.6%)	48.90%	17851404343	17503452507 (98.05%)	16836240818 (94.31%)
J04M-1	109276042	107087522 (98%)	47.25%	15798808477	15526821931 (98.28%)	14994738826 (94.91%)
J04M-2	137967834	131611748 (95.39%)	46.53%	18713013149	18007258920 (96.23%)	16748202673 (89.50%)
J04M-3	151333842	145627326 (96.23%)	47.86%	20773772202	20029218376 (96.42%)	18680077603 (89.92%)
J05M-1	131613502	125900828 (95.66%)	47.36%	17906681937	17231920337 (96.23%)	16025801209 (89.50%)
J05M-2	133834768	128208156 (95.8%)	48.30%	18230865699	17548226239 (96.26%)	16328197015 (89.56%)
J05M-3	99790248	95543382 (95.74%)	47.20%	13602759854	13100318985 (96.31%)	12200732031 (89.69%)
Total	2289564890	2207813200		319366117305		

Table S2. GO enrichment analysis of DEGs in Cellular Component.

Stages	Go ID	Description	Gene Number	FDR
I [1]	GO:0031224	intrinsic component of membrane	259	1.54E-11
	GO:0016020	membrane	361	2.94E-09
	GO:0044425	membrane part	269	5.81E-08
	GO:0030312	external encapsulating structure	41	0.000195606
	GO:0071944	cell periphery	43	0.000350184
II	GO:0031224	intrinsic component of membrane	85	1.84E-14
	GO:0030312	external encapsulating structure	18	2.45E-12
	GO:0016020	membrane	120	8.83E-10
	GO:0044425	membrane part	91	0.00142213
	GO:0071944	cell periphery	18	0.002663931
III	GO:0016021	integral component of membrane	5	0.011316755
	GO:0031224	intrinsic component of membrane	40	0.011316755
	GO:0044434	chloroplast part	10	0.016488573
	GO:0044425	membrane part	42	0.016488573
	GO:0009507	chloroplast	10	0.016488573
	GO:0044435	plastid part	18	0.029140896
IV	GO:0005737	cytoplasm	35	0.001703956
	GO:0044444	cytoplasmic part	33	0.004101946
	GO:0005623	cell	54	0.013077092
	GO:0044464	cell part	54	0.013077092
	GO:0009536	plastid	21	0.014961503
	GO:0005576	extracellular region	5	0.015502068
	GO:0005622	intracellular	51	0.032508287
V	GO:0005737	cytoplasm	36	2.15E-08
	GO:0044444	cytoplasmic part	34	2.21E-07
	GO:0005623	cell	46	1.83E-05
	GO:0044464	cell part	46	1.83E-05
	GO:0044424	intracellular part	45	1.92E-05
	GO:0005622	intracellular	45	2.29E-05
	GO:0009536	plastid	22	4.00E-05
	GO:0043229	intracellular organelle	38	0.000369746
	GO:0043231	intracellular membrane-bounded organelle	35	0.000572207
	GO:0043226	organelle	38	0.000572207
	GO:0043227	membrane-bounded organelle	35	0.001166744

[1] Stage I: 0 d vs 7 d, Stage II: 7 d vs 14 d, Stage III: 14 d vs 21 d, Stage IV: 21 d vs 28 d, Stage V: 28 d vs 35 d.

Table S3. GO enrichment analysis of DEGs in Molecular Function.

Stages	Go ID	Description	Gene Number	FDR
I [1]	GO:0016491	oxidoreductase activity	178	0.006002521
	GO:0016639	oxidoreductase activity, acting on the CH-NH2 group of donors, NAD or NADP as acceptor	6	0.006002521
	GO:0004672	protein kinase activity	127	0.006588417
	GO:0016773	phosphotransferase activity, alcohol group as acceptor	133	0.006588417
	GO:0016301	kinase activity	160	0.00998398
	GO:0016705	oxidoreductase activity, acting on paired donors, with incorporation or reduction of molecular oxygen	29	0.014582235
	GO:0004553	hydrolase activity, hydrolyzing O-glycosyl compounds	37	0.016140925
	GO:0015291	secondary active transmembrane transporter activity	21	0.018281468
	GO:0046906	tetrapyrrole binding	46	0.023845197
	GO:0016798	hydrolase activity, acting on glycosyl bonds	45	0.023845197
	GO:0015926	glucosidase activity	14	0.027667152
	GO:0043169	cation binding	276	0.027667152
	GO:0032553	ribonucleotide binding	10	0.030994308
	GO:0005506	iron ion binding	4	0.034489888
	GO:0016679	oxidoreductase activity, acting on diphenols and related substances as donors	10	0.034489888
	GO:0016682	oxidoreductase activity, acting on diphenols and related substances as donors, oxygen as acceptor	10	0.034489888
	GO:0052592	oxidoreductase activity, acting on CH or CH2 groups, with an iron-sulfur protein as acceptor	5	0.034489888
	GO:0045181	glutamate synthase activity, NAD(P)H as acceptor	3	0.047983581
II	GO:0016706	oxidoreductase activity, acting on paired donors, with incorporation or reduction of molecular oxygen, 2-oxoglutarate as one donor, and incorporation of one atom each of oxygen into both donors	6	0.011675432
	GO:0051213	dioxygenase activity	6	0.024158305
III	GO:0016651	oxidoreductase activity, acting on NAD(P)H	9	0.009442423
	GO:0016491	oxidoreductase activity	35	0.019997842
	GO:0048037	cofactor binding	9	0.026131361
	GO:0046906	tetrapyrrole binding	12	0.028499899
	GO:0016679	oxidoreductase activity, acting on diphenols and related substances as donors	4	0.039263091
	GO:0016682	oxidoreductase activity, acting on diphenols and related substances as donors, oxygen as acceptor	4	0.039263091
IV	GO:0016491	oxidoreductase activity	24	0.003945661
V	GO:0016491	oxidoreductase activity	21	0.006166828
	GO:0000104	succinate dehydrogenase activity	2	0.041394045

[1] Stage I: 0 d vs 7 d, Stage II: 7 d vs 14 d, Stage III: 14 d vs 21 d, Stage IV: 21 d vs 28 d, Stage V: 28 d vs 35 d.

Table S4. GO enrichment analysis of DEGs in Biological Process.

Stages	Go ID	Description	Gene Number	FDR
I [1]	GO:0006595	polyamine metabolic process	9	0.049998197
II	N/A			
III	N/A			
IV	N/A			
V	GO:0044281	small molecule metabolic process	24	0.021550796
	GO:0044283	small molecule biosynthetic process	9	0.03611903
	GO:0006101	citrate metabolic process	4	0.03611903
	GO:0072350	tricarboxylic acid metabolic process	4	0.03611903
	GO:0043436	oxoacid metabolic process	17	0.036879098
	GO:0006082	organic acid metabolic process	17	0.036879098

[1] Stage I: 0 d vs 7 d, Stage II: 7 d vs 14 d, Stage III: 14 d vs 21 d, Stage IV: 21 d vs 28 d, Stage V: 28 d vs 35 d.

Table S5. KEGG enrichment analysis of DEGs.

Stages	Pathway ID	Pathway	Gene Number	FDR
I [1]	ko04075	Plant hormone signal transduction	75	7.67E-06
	ko00940	Phenylpropanoid biosynthesis	46	3.24E-04
	ko00900	Terpenoid backbone biosynthesis	29	3.24E-04
	ko00592	alpha-Linolenic acid metabolism	28	5.84E-04
	ko00909	Sesquiterpenoid and triterpenoid biosynthesis	12	5.84E-04
	ko00910	Nitrogen metabolism	22	1.26E-03
	ko00906	Carotenoid biosynthesis	16	2.41E-03
	ko00514	Other types of O-glycan biosynthesis	10	9.67E-03
	ko00072	Synthesis and degradation of ketone bodies	6	1.06E-02
	ko00905	Brassinosteroid biosynthesis	8	2.83E-02
	ko04146	Peroxisome	33	2.83E-02
	ko04626	Plant-pathogen interaction	51	2.93E-02
	ko00071	Fatty acid degradation	24	2.93E-02
	ko00500	Starch and sucrose metabolism	61	3.55E-02
	ko00052	Galactose metabolism	20	3.90E-02
	ko00100	Steroid biosynthesis	15	4.73E-02
	ko04712	Circadian rhythm - plant	15	4.73E-02
II	ko00940	Phenylpropanoid biosynthesis	24	7.67E-05
	ko00906	Carotenoid biosynthesis	10	8.20E-04
	ko00500	Starch and sucrose metabolism	27	3.23E-02
	ko00520	Amino sugar and nucleotide sugar metabolism	18	4.55E-02
	ko00903	Limonene and pinene degradation	4	4.62E-02
	ko00040	Pentose and glucuronate interconversions	13	4.62E-02
III	ko00909	Sesquiterpenoid and triterpenoid biosynthesis	7	1.03E-05
	ko00940	Phenylpropanoid biosynthesis	14	1.17E-04
	ko00072	Synthesis and degradation of ketone bodies	4	4.62E-04
	ko00900	Terpenoid backbone biosynthesis	9	6.85E-04
	ko04626	Plant-pathogen interaction	15	8.24E-04
	ko00100	Steroid biosynthesis	6	6.11E-03
	ko00280	Valine, leucine and isoleucine degradation	8	1.37E-02
	ko00650	Butanoate metabolism	4	3.86E-02
IV	ko01200	Carbon metabolism	33	8.53E-07
	ko01230	Biosynthesis of amino acids	28	6.22E-06
	ko00020	Citrate cycle (TCA cycle)	13	1.26E-04
	ko00630	Glyoxylate and dicarboxylate metabolism	12	6.07E-04
	ko00620	Pyruvate metabolism	14	6.07E-04
	ko01210	2-Oxocarboxylic acid metabolism	10	1.91E-03
	ko00710	Carbon fixation in photosynthetic organisms	10	4.70E-03
	ko00030	Pentose phosphate pathway	8	8.30E-03
	ko00290	Valine, leucine and isoleucine biosynthesis	5	1.10E-02
V	ko01200	Carbon metabolism	35	1.65E-09
	ko01230	Biosynthesis of amino acids	28	4.22E-07
	ko00020	Citrate cycle (TCA cycle)	13	3.23E-05
	ko00620	Pyruvate metabolism	14	1.93E-04
	ko00710	Carbon fixation in photosynthetic organisms	10	2.45E-03
	ko01210	2-Oxocarboxylic acid metabolism	9	3.61E-03
	ko00030	Pentose phosphate pathway	8	4.19E-03
	ko00010	Glycolysis / Gluconeogenesis	12	6.22E-03
	ko00290	Valine, leucine and isoleucine biosynthesis	5	6.28E-03

ko00630	Glyoxylate and dicarboxylate metabolism	9	8.26E-03
ko01220	Degradation of aromatic compounds	3	1.29E-02
ko00920	Sulfur metabolism	6	2.27E-02
ko00230	Purine metabolism	13	2.44E-02
ko00400	Phenylalanine, tyrosine and tryptophan biosynthesis	5	4.57E-02

[1] Stage I: 0 d vs 7 d, Stage II: 7 d vs 14 d, Stage III: 14 d vs 21 d, Stage IV: 21 d vs 28 d, Stage V: 28 d vs 35 d.

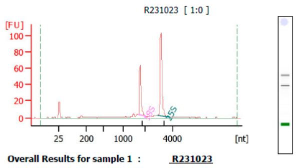

Overall Results for sample 1 : R231023

RNA Area: 227.2
RNA Concentration: 171 ng/µl
rRNA Ratio [25s / 18s]: 1.8
RNA Integrity Number (RIN): 9.6 (B.02.08)
Result Flagging Color:
Result Flagging Label: RIN: 9.60

Fragment table for sample 1 : R231023

Name	Start Size [nt]	End Size [nt]	Area	% of total Area
18S	1,694	2,077	53.8	23.7
25S	2,853	3,831	98.9	43.5

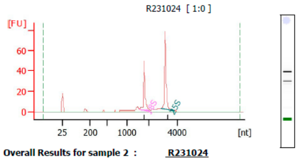

Overall Results for sample 2 : R231024

RNA Area: 182.5
RNA Concentration: 137 ng/µl
rRNA Ratio [25s / 18s]: 1.5
RNA Integrity Number (RIN): 9.7 (B.02.08)
Result Flagging Color:
Result Flagging Label: RIN: 9.70

Fragment table for sample 2 : R231024

Name	Start Size [nt]	End Size [nt]	Area	% of total Area
18S	1,545	2,067	47.5	26.0
25S	2,838	3,824	73.4	40.2

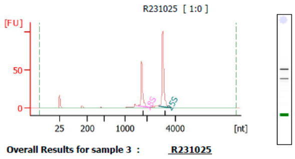

Overall Results for sample 3 : R231025

RNA Area: 220.4
RNA Concentration: 165 ng/µl
rRNA Ratio [25s / 18s]: 1.7
RNA Integrity Number (RIN): 9.8 (B.02.08)
Result Flagging Color:
Result Flagging Label: RIN: 9.80

Fragment table for sample 3 : R231025

Name	Start Size [nt]	End Size [nt]	Area	% of total Area
18S	1,552	2,100	57.6	26.1
25S	2,795	3,744	96.0	43.6

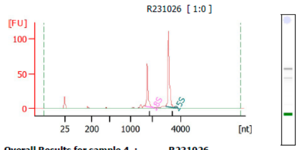

Overall Results for sample 4 : R231026

RNA Area: 228.2
RNA Concentration: 171 ng/µl
rRNA Ratio [25s / 18s]: 1.9
RNA Integrity Number (RIN): 9.5 (B.02.08)
Result Flagging Color:
Result Flagging Label: RIN: 9.50

Fragment table for sample 4 : R231026

Name	Start Size [nt]	End Size [nt]	Area	% of total Area
18S	1,691	2,048	52.7	23.1
25S	2,893	3,864	99.6	43.6

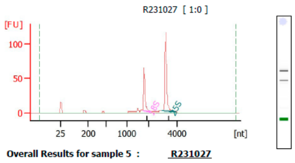

Overall Results for sample 5 : R231027

RNA Area: 224.7
RNA Concentration: 169 ng/µl
rRNA Ratio [25s / 18s]: 1.9
RNA Integrity Number (RIN): 9.6 (B.02.08)
Result Flagging Color:
Result Flagging Label: RIN: 9.60

Fragment table for sample 5 : R231027

Name	Start Size [nt]	End Size [nt]	Area	% of total Area
18S	1,714	2,136	53.8	24.0
25S	2,906	3,913	103.9	46.2

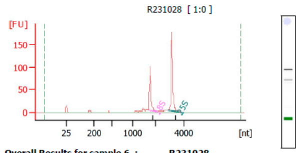

Overall Results for sample 6 : R231028

RNA Area: 345.5
RNA Concentration: 259 ng/µl
rRNA Ratio [25s / 18s]: 1.9
RNA Integrity Number (RIN): 9.6 (B.02.08)
Result Flagging Color:
Result Flagging Label: RIN: 9.60

Fragment table for sample 6 : R231028

Name	Start Size [nt]	End Size [nt]	Area	% of total Area
18S	1,700	2,098	84.5	24.5
25S	2,863	3,948	161.8	46.8

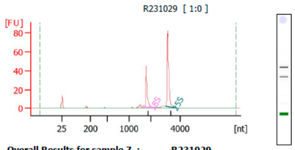

Overall Results for sample 7 : R231029

RNA Area: 157.8
RNA Concentration: 118 ng/µl
rRNA Ratio [25s / 18s]: 1.8
RNA Integrity Number (RIN): 9.8 (B.02.08)
Result Flagging Color:
Result Flagging Label: RIN: 9.80

Fragment table for sample 7 : R231029

Name	Start Size [nt]	End Size [nt]	Area	% of total Area
18S	1,598	2,211	41.0	26.0
25S	2,959	3,804	72.3	45.8

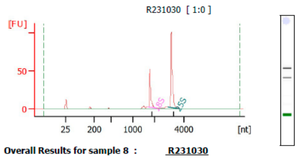

Overall Results for sample 8 : R231030

RNA Area: 184.0
RNA Concentration: 138 ng/µl
rRNA Ratio [25s / 18s]: 2.0
RNA Integrity Number (RIN): 9.5 (B.02.08)
Result Flagging Color:
Result Flagging Label: RIN: 9.50

Fragment table for sample 8 : R231030

Name	Start Size [nt]	End Size [nt]	Area	% of total Area
18S	1,703	2,086	44.2	24.0
25S	2,917	3,790	88.0	47.8

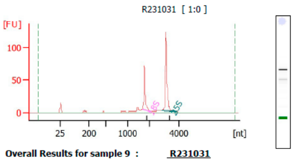

Overall Results for sample 9 : R231031

RNA Area: 257.9
RNA Concentration: 194 ng/µl
rRNA Ratio [25s / 18s]: 1.9
RNA Integrity Number (RIN): 9.5 (B.02.08)
Result Flagging Color:
Result Flagging Label: RIN: 9.50

Fragment table for sample 9 : R231031

Name	Start Size [nt]	End Size [nt]	Area	% of total Area
18S	1,686	2,058	61.1	23.7
25S	2,834	3,860	114.9	44.5

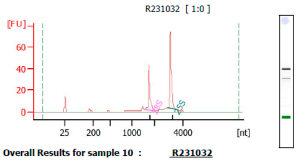

Overall Results for sample 10 : R231032

RNA Area: 172.4
RNA Concentration: 129 ng/µl
rRNA Ratio [25s / 18s]: 1.5
RNA Integrity Number (RIN): 9.5 (B.02.08)
Result Flagging Color:
Result Flagging Label: RIN: 9.50

Fragment table for sample 10 : R231032

Name	Start Size [nt]	End Size [nt]	Area	% of total Area
18S	1,574	2,101	42.7	24.8
25S	2,959	3,567	62.8	36.4

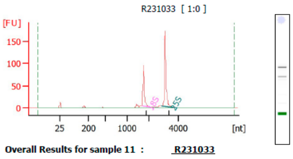

Overall Results for sample 11 : R231033

RNA Area: 342.7
RNA Concentration: 257 ng/µl
rRNA Ratio [25s / 18s]: 1.9
RNA Integrity Number (RIN): 9.6 (B.02.08)
Result Flagging Color:
Result Flagging Label: RIN: 9.60

Fragment table for sample 11 : R231033

Name	Start Size [nt]	End Size [nt]	Area	% of total Area
18S	1,677	2,058	82.2	24.0
25S	2,820	3,943	159.2	46.4

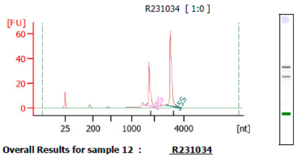

Overall Results for sample 12 : R231034

RNA Area: 155.9
RNA Concentration: 117 ng/µl
rRNA Ratio [25s / 18s]: 1.5
RNA Integrity Number (RIN): 9.4 (B.02.08)
Result Flagging Color:
Result Flagging Label: RIN: 9.40

Fragment table for sample 12 : R231034

Name	Start Size [nt]	End Size [nt]	Area	% of total Area
18S	1,558	2,116	38.5	24.7
25S	2,792	3,855	58.8	37.7

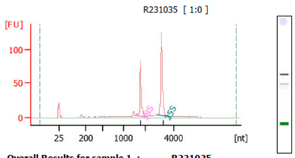

Overall Results for sample 1 : R231035

RNA Area: 274.7
RNA Concentration: 191 ng/µl
rRNA Ratio [25s / 18s]: 1.7
RNA Integrity Number (RIN): 10 (B.02.08)
Result Flagging Color:
Result Flagging Label: RIN:10

Fragment table for sample 1 : R231035

Name	Start Size [nt]	End Size [nt]	Area	% of total Area
18S	1,543	2,075	76.6	27.9
25S	2,779	3,806	126.6	46.1

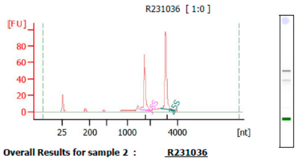

Overall Results for sample 2 : R231036

RNA Area: 232.4
RNA Concentration: 161 ng/µl
rRNA Ratio [25s / 18s]: 1.5
RNA Integrity Number (RIN): 9.9 (B.02.08)
Result Flagging Color:
Result Flagging Label: RIN: 9.90

Fragment table for sample 2 : R231036

Name	Start Size [nt]	End Size [nt]	Area	% of total Area
18S	1,549	2,093	62.9	27.1
25S	2,792	3,799	96.6	41.6

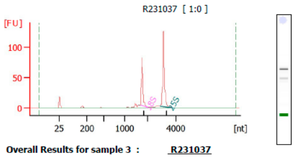

Overall Results for sample 3 : R231037

RNA Area: 282.6
RNA Concentration: 196 ng/µl
rRNA Ratio [25s / 18s]: 1.8
RNA Integrity Number (RIN): 9.6 (B.02.08)
Result Flagging Color:
Result Flagging Label: RIN: 9.60

Fragment table for sample 3 : R231037

Name	Start Size [nt]	End Size [nt]	Area	% of total Area
18S	1,706	2,027	66.5	23.5
25S	2,875	3,820	122.4	43.3

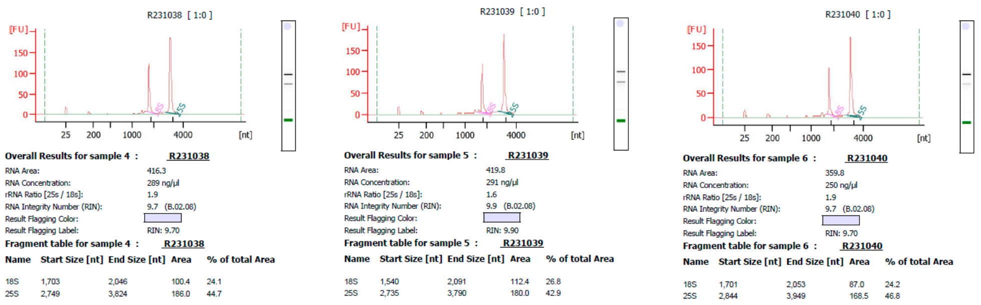

Figure S1. The quality of RNA samples.

© 2018 by the authors. Licensee MDPI, Basel, Switzerland. This article is an open access article distributed under the terms and conditions of the Creative Commons Attribution (CC BY) license (http://creativecommons.org/licenses/by/4.0/).
